# Early assessment of extremity compartment syndrome by biochemical markers in a rat model

**DOI:** 10.55730/1300-0144.5552

**Published:** 2022-10-10

**Authors:** Ahmet YILDIRIM, Özkan ÖNAL, Zeliha Esin ÇELİK, Hüsamettin VATANSEV, Esra PAYDAŞ HATAYSAL

**Affiliations:** 1 Department of Orthopedics and Traumatology, Medova Private Hospital, Konya, Turkey; 2 Department of Anesthesiology and Reanimation, Faculty of Medicine, Selçuk University, Konya, Turkey; 3 Department of Pathology, Faculty of Medicine, Selçuk University, Konya, Turkey; 4 Department of Biochemistry, Faculty of Medicine, Selçuk University, Konya, Turkey; 5 Department of Biochemistry, Göztepe Prof. Dr. Süleyman Yalçın City Hospital, İstanbul, Turkey

**Keywords:** Compartment syndrome, ischemia, biochemical markers, neopterin, diagnostic techniques, trauma

## Abstract

**Background/aim:**

This experimental study aimed to define a biochemical marker that will enable early diagnosis of acute compartment syndrome (ACS) of extremities, a mortal condition that occurs due to trauma.

**Materials and methods:**

A total of 15 Wistar rats were included in the study in which saline infusion technique, a clinically compatible ACS model, was applied. After the rats were anesthetized with ketamine-xylazine, the in-compartment pressure of the hind limb was slowly increased with saline delivered through the angiocatheter, and after reaching the target compartment pressure, the pressure level was kept with a rubber tourniquet. The in-compartment pressure level was continuously monitored with a pressure transducer. The rats were divided into three groups. No intervention was applied to the control group (CG) (n = 3). In study group 1 (SG1) (n = 6), ACS was created using the saline infusion technique, keeping the in-compartment pressure between 30 and 40 mmHg for 45 min. In study group 2 (SG2) (n = 6), ACS was created using the saline infusion technique, keeping the in-compartment pressure between 30 and 40 mmHg for 90 min. Fasciotomy was performed on all rats. Tissue samples were obtained for histopathological examination and blood samples for biochemical analysis.

**Results:**

Total oxidant status (TOS) (*p* = 0.004), ischemia-modified albumin (IMA) (p = 0.030), aspartate transferase (AST) (*p* = 0.003) and neopterin (*p* = 0.012) levels differed significantly between groups in the early period of muscle ischemia. In fact, TOS levels differed significantly between the groups even in the cellular phase where signs of ischemia were not observed (*p* = 0.048, *p =* 0.024). According to histopathological evaluation, there was no significant difference between the groups.

**Conclusion:**

TOS can be detected in the early reversible stage of ischemia, when the histopathological findings of ACS do not occur.

## 1. Introduction

Acute compartment syndrome (ACS) is a complication most commonly caused by the trauma of the extremities; however, it may also be observed in conditions that cause bleeding, edema, or perfusion in any myofascial compartment of the body [1,2]. Blood and fluid accumulation due to fracture, crush, or ischemia in the extremity increases the myofascial compartment mass. The increased mass effect causes an increase in pressure within the compartment as the fascia and other muscles connective tissues are not flexible. This pressure increase causes venous hypertension [3], and progressive tissue ischemia by putting pressure on thin-walled vessels. Cell death begins after ischemia and osmotically active cellular contents of lysed cells fill the interstitial space, causing more fluid to accumulate and further increase in compartment pressure [4]. Arteriolar perfusion also deteriorates, and microvascular collapse occurs [5]. Myonecrosis is seen in the third hour of ACS due to injury [6]. Irreversible ischemic damage occurs in the myoneural tissues within the muscle compartment 6 h after the circulatory impairment develops [4].

The only effective treatment for ACS is emergency surgical fasciotomy in the early period. The ideal time for fasciotomy is at the latest 6 h after injury. It is not recommended to perform fasciotomy 36 h after the injury as the rate of normal limb function restoration decreases to 8% even after 12 h [7,8].

Although it is considered an emergency, it is highly difficult to diagnose ACS in the early period since the current diagnosis is based on the continuous measurement of the in-compartmental pressure (ICP) along with the patient’s clinical findings.

The accepted clinical manifestations of ACS are pain caused by passive stretching of the related muscle which is so severe that it is disproportional to the injury and paresthesia in the distribution of any sensory nerve in the compartment. However, the use of pain cannot be used alone as a screening test as the clinical signs and symptoms of patients with exposure to trauma and ACS can change instantly due to trauma [9] because while pain may occur with passive stretching in the early period, it may not be felt at all in advanced ACS. Additionally, the pain may feel like a burning sensation at the early stage and the clinician can have difficulty diagnosing the condition [7].

The continuous measurement of ICP, which is the other cornerstone of ACS diagnosis, is difficult and subjective. Hence, it is challenging to diagnose ACS without mistakes in the early period. Measuring the in-compartment pressure higher than 30 mmHg can be used as a threshold for the diagnosis of ACS since compartment pressure is less than 10 mmHg under normal conditions. However, having a single normal ICP does not exclude acute compartment syndrome and continuous measurement is required. Perfusion pressure affected by the diastolic pressure value of the patient also plays a role in the emergence of ACS [7]. Moreover, the pressure sensor should be placed in an area around 5 cm near the fracture line where there is no direct contact with the fracture to measure the ICP correctly [10]. Different severity of muscle damage changes the ICP measurement [11]. Thus, a high rate of false-positive measurement of pressure measurements and unnecessary fasciotomy decisions are other problems. Different pressure thresholds are also recommended for fasciotomy indication and diagnosis of ACS becomes more difficult [12–14].

All these reasons necessitate studies to find noninvasive methods with high sensitivity and specificity that can objectively detect tissue ischemia in the early period before myonecrosis is observed. The availability of such a method will help the clinician decide whether to operate on a patient with high ICP before irreversible damage occurs in the early stage of ischemia. We conducted this study hypothesizing that biochemical markers IMA, TAS, TOS, ALT, AST, CK, and neopterin can diagnose acute compartment syndrome in the early period before muscle necrosis develops and are more sensitive and specific than pressure readings.

## 2. Materials and methods

The study was conducted following the approval of the Ethics Committee from the Animal Care Committee at Selçuk University Experimental Research Center (2018/10) and in accordance with the European Community guidelines and Helsinki and Tokyo Declarations. Fifteen male Wistar rats with the same physiological and biological characteristics included in the study could access food and water ad libitum during their shelter. The number of rats included and the applied ACS model were determined based on the reference article [15].

### 2.1. Experimental groups and experimental model

**Figure 1:**
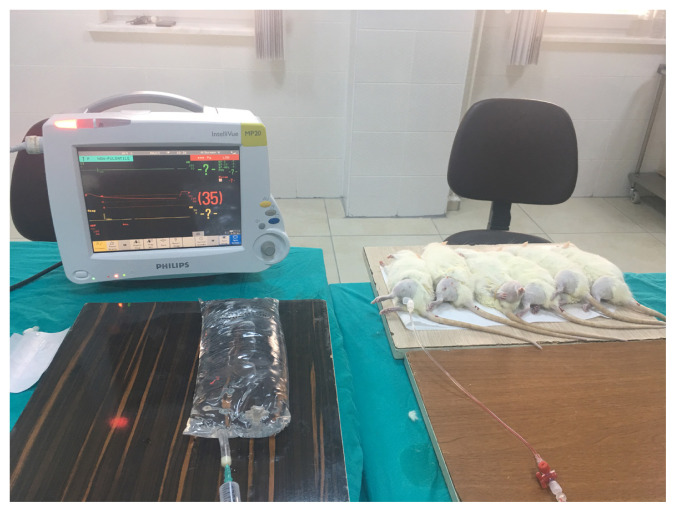
The image of isotonic saline injection via an 18-gauge angiocatheter into the anterior compartment of the left hind limb in the study groups.

**Figure 2:**
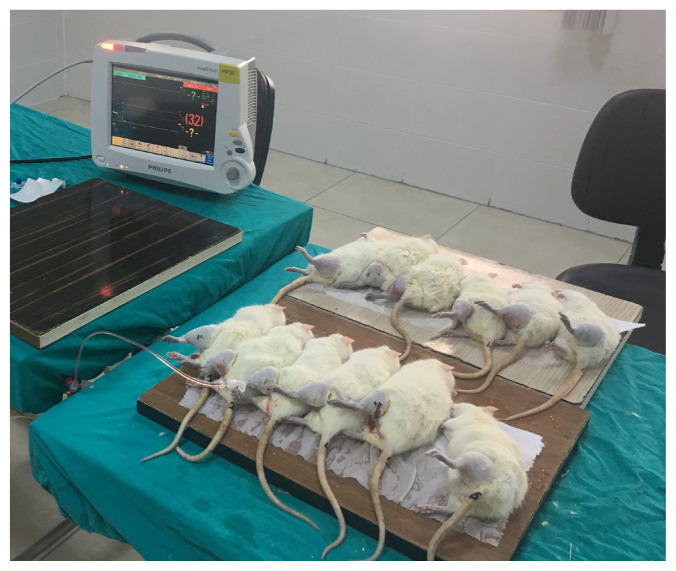
The image of extremity compartment syndrome that was created using a rubber band tourniquet with standard compression.

Fifteen male rats (250–300 g) were anesthetized with 65 mg/kg intraperitoneal ketamine (Ketalar; Eczacıbaşı, İstanbul, Turkey) and 7 mg/kg xylazine hydrochloride (Rompun; Bayer, Leverkusen, Germany) to maintain general anesthesia. The compartment pressure was increased by gradually infusing isotonic saline via an 18-gauge angiocatheter into the anterior compartment of the left hind limb in the study groups (Figure 1) and maintained using a rubber band tourniquet with a standard compression (Figure 2). An electronic pressure monitoring system (Philips IntelliVue MP 20) and tissue transducer through an angiocatheter were used to monitor the compartment pressure between 30 and 40 mmHg. There were three rat groups. The first group was the control group (n = 3) with similar characteristics to study groups to study the blood samples and determine basic serum levels of ischemic tissue markers. No saline was infused into the compartment of the control group (CG). We formed two study groups (SG) to analyze the effect of high ICP on tissue perfusion and ischemic damage over two different time periods. We observed the first study group (SG1) for 45 min before taking blood and tissue samples after saline injection to the compartment to see the early effects of ACS and waited for 90 min for the same procedure in the second study group (SG2) to observe the late effects of ACS.

**Figure 3:**
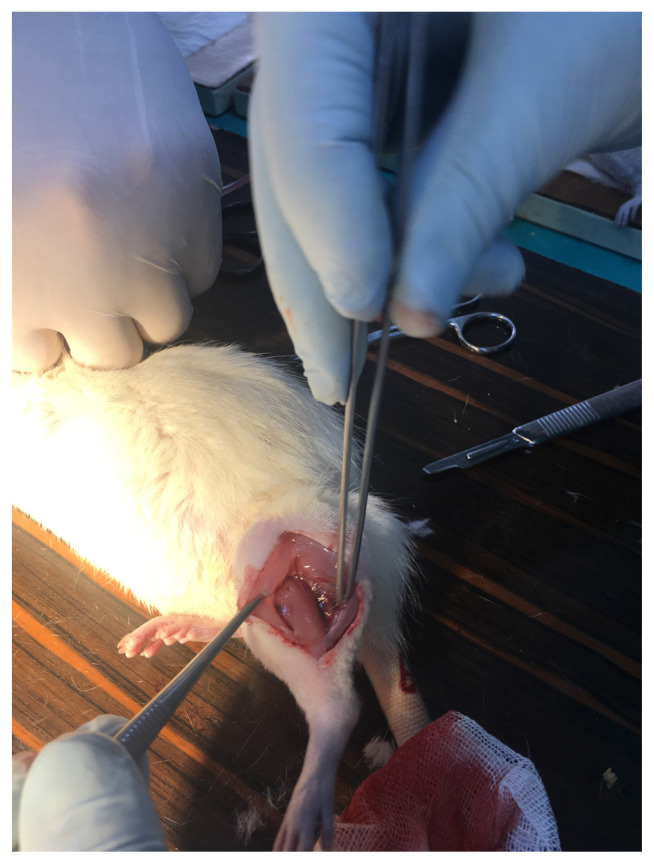
The image of extensor digitorum longus muscle extraction.

**Figure 4:**
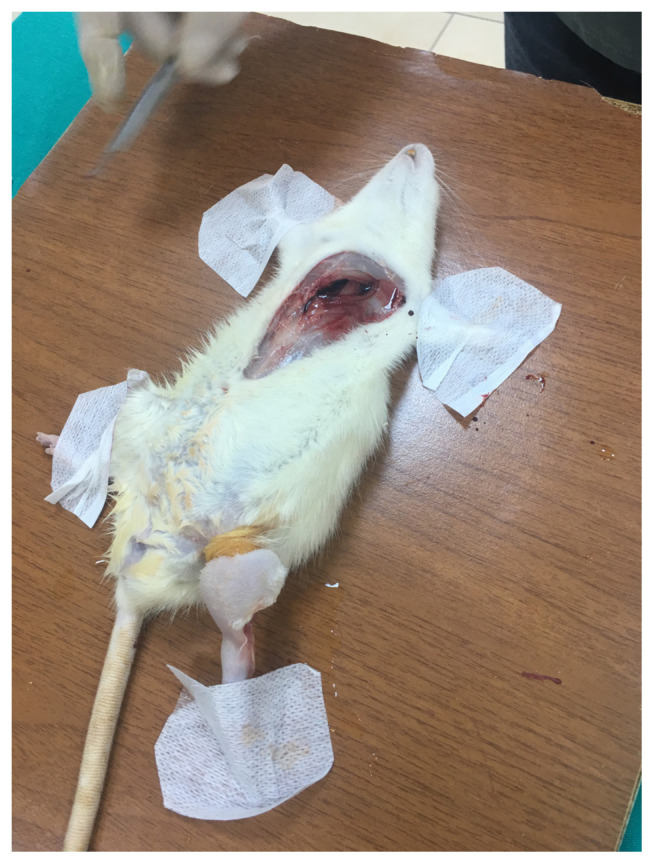
The sample image of the rats’ sacrification process.

The extensor digitorum longus (EDL) muscle was extracted to observe pathologic changes of the tissue at the end of 45 min for SG1 and 90 min for SG2 after the compartment procedure began. The muscle preparation for the extraction was described in the literature [15,16]. We detached the biceps femoris to expose the tibialis anterior and the lateral gastrocnemius muscles. The tibialis anterior and the lateral gastrocnemius muscles were separated to expose the EDL and the overlying fascia. The EDL tendon was cut to extract the whole EDL muscle (Figure 3). After the extraction of EDL, all the rats were sacrificed by drawing blood from their heart (Figure 4) and these blood samples were collected directly into serum separator tubes (VACUETTE, Greiner Bio-One GmbH, Kremsmünster, Austria).

### 2.2. Histopathologic examination

Tissue samples taken for pathological examination were fixed in 10% neutral buffered formaldehyde solution for 24 h and embedded in paraffin. These samples were evaluated microscopically after taking 4-μm-thick sections with the help of microtome and staining with hematoxylin-eosin dye. Images were acquired with Leica LX53 light microscope. The presence of edema, inflammation, and necrosis in tissues and the universality of these parameters were assessed for each group by modeling the reference articles [17,18]. Tissue samples were scored from 0 to 3. If there were no pathological findings, the score was 0. If the parameters were observed on a mild level, the score was 1; the medium level was 2; and the severe level was 3.

### 2.3. Laboratory analysis

Centrifugation process at 1500 × *g* for 10 min was performed after the blood samples were taken to the test tubes. Serum was separated, pipetted into Eppendorf tubes, and stored frozen at −80 °C until analysis. Biochemical serum parameters sensitive to ischemia, IMA, TAS, TOS, CK, AST, ALT, and neopterin were measured in all rats.

The AST, ALT, and CK enzyme activities were analyzed with a Beckman Coulter Au5800 autoanalyzer (Beckman Coulter Inc, Brea, CA, USA) at the Biochemistry Laboratory of Selçuk University Faculty of Medicine.

Serum values of TAS and TOS were determined using the automated measurement (Rel Assay Diagnostics kit, Mega Tıp, Gaziantep, Turkey) and Erel’s colorimetric methods [19].

Serum values of IMA were measured using an ELISA kit (Elabscience Rat IMA ELISA kit (catalog number: E-EL-R2486) according to the manufacturer’s protocol. The detection range of the kit was 6.25–400 ng/mL and the sensitivity was 3.75 ng/mL.

Neopterin levels were measured by Thermo Scientific Ultimate 3000 Ultra Performance Liquid Chromatography (UPLC). Serum neopterin levels were measured as described by Mahendra L et al. [20]. Separations were carried out with a Phenomenex C18 column (50 mm × 4.6 mm, part no: 00B-4041-E0). The flow rate of 1.5 mL/min with water: Acetonitrile (99:1 v/v) as the mobile phase and the fluorescence detector was set at 353 nm of excitation and 438 nm of emission for detection.

### 2.4. Data analysis

Data were analyzed using the SPSS 25.0 package software program (IBM, Armonk, NY). The Mann–Whitney U test was used for the abnormally distributed variables. Continuous variables were analyzed with the Kruskal–Wallis H test. Data were analyzed using the Kolmogorov–Smirnov normality test. The sample size was determined according to the importance of the 3R (reduce, refine, and replace) rule for scientific experiments involving animals and old studies using a similar rat model [15,17,18,21]. All analyses were performed with 95% confidence intervals. A p-value of <0.05 was considered significant. All data were expressed in mean ± standard error (SE).

## 3. Results

**Figure 5:**
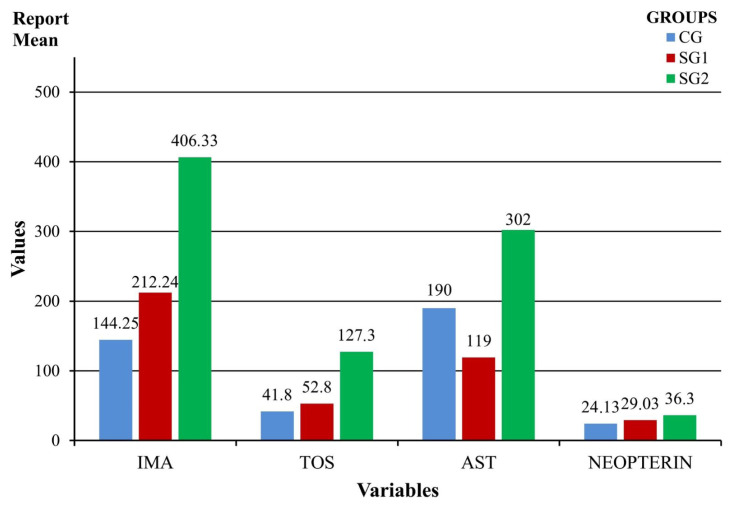
The comparison of IMA, TOS, AST, and neopterin values between study groups.

Seven biochemical markers sensitive to ischemia were assessed and TOS, IMA, AST, and neopterin were valuable in demonstrating early limb muscle ischemia TOS was the earliest predictive marker of ACS, but ALT, CK, and TAS changes were insignificant (Table) (Figure 5).

Mean IMA levels differed significantly between groups (*p* = 0.030). IMA values were 144.25 ± 31.72 ng/mL in the CG, 212.24 ± 155.42 ng/mL in the SG1, and 406.33 ± 248.53 ng/mL in SG2. There was a significant difference between the two groups (*p* = 0.020).

Mean TOS levels differed significantly between groups (*p =* 0.004). TOS value was 41.08 ± 4.06 μmol/L in the CG, 52.80 ± 15.45 μmol/L in SG1, and 127.30 ± 30.07 μmol/L in SG2. There was a significant difference between CG and SG1 (*p =* 0.048) and CG and SG2 (*p =* 0.024) in terms of TOS levels. TOS was found to be susceptible to limb muscle ischemia without any histopathological changes in the early stage of ischemia.

There was a significant difference between the groups in terms of mean AST level (*p =* 0.003). AST level was 170.00 ± 40.85 U/L in the CG, 119 ± 20.07 U/L, and 464.66 ± 334.77 U/L in SG2. There was a significant difference between the two groups (*p =* 0.021).

There was a significant difference between the groups in terms of mean neopterin levels (*p =* 0.012). Neopterin levels were 24.66 ± 3.00 ng/mL in CG, 29.03 ±4.96 ng/mL in SG1, and 434.94 ± 3.83 ng/mL in SG2. There was a significant difference between the two groups (*p =* 0.016).

**Figure 6:**
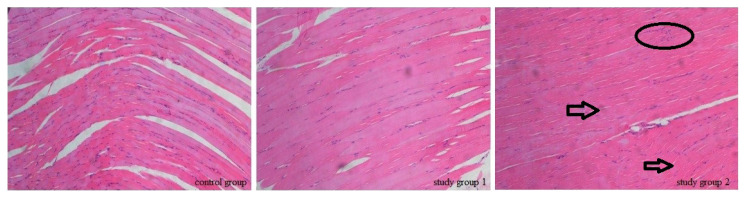
In study group 2, minimal tissue edema resulted in a mild separation between muscle fibers (arrows) and to “mild muscle fibers (circle) inflammation.

According to the histopathological evaluation, the score of all samples in CG and SG1 was evaluated as 0. While the score of 2 samples in SG2 was evaluated as 0, some minor pathological changes such as edema and inflammation occurred in four samples of SG2, and their score was evaluated as 1 (Figure 6).

## 4. Discussion

The diagnosis of ACS is based on clinical findings. However, the diagnosis of ACS is not completely possible due to the momentary change in the findings of the patients. Especially due to trauma and problems caused by trauma or the clinical examination of pediatric patients cannot be performed objectively [22]. Failure to diagnose on time causes a delay in fasciotomy. Late fasciotomy causes severe muscle fibrosis, limb contractures, and a 10-fold increase in complication rates (4.5% vs. 54%) [8]. The clinician needs objective data to confirm ACS. Although several diagnostic tests have been proposed, no sensitive and specific markers have been discovered.

According to the results of our study, the biochemical markers IMA, TOS, TAS, ALT, AST, CK, the neopterin values measured at the 90th min of the compartment pressure applied high enough to cause ACS were clinically significant compared to the control group. Moreover, unlike other markers, the TOS value increased significantly even in the very early phase of the high-pressure application, namely in the first 45 min. Our histopathological findings did not differ between the control group and the study group because it is known that the histopathological findings of ACS at the nucleus level occur not in the early period but after the 3rd hour [23].

Ultrastructural evidence of irreversible damage to the muscle cell occurs in 3 to 4 h after ischemia begins. At this stage, mitochondria swell, nuclei become denser, and myofilaments degenerate. Endothelial cells degenerate at this stage and the capillaries that feed the muscle are damaged [23]. Nucleotide degeneration in muscle cells is seen 3 h after ischemia begins [24–26]. Histological and significant changes occur 6 to 8 h after the onset of ischemia [27–29].

In addition to high compartment pressure, ischemia-reperfusion injury also plays an important role in the physiopathology of ACS. TOS and TAC levels have been examined in studies conducted to detect ischemia-reperfusion injury, which is a very important clinical problem, and it has been shown that these markers are helpful both in the diagnosis of ischemia-reperfusion (IR) injury and in the investigation of the treatment of IR injury with various agents [30–32]. In our study, TOS values were found to be high both at the 45th and 90th minutes of the increased ICP created to create ACS. The fact that the TOS value was determined to be high even at the 45th minute of the ICP showed that ACS can be detected at a very early stage, where it is impossible to detect both histopathological and biochemical markers.

Inflammatory response markers such as white blood cell count (WBC), erythrocyte sedimentation rate (ESR) and C-reactive protein (CRP) are increased after any limb is exposed to trauma.

Once ACS develops, creatine kinase and lactate levels rise due to muscle crash and anaerobic metabolism, respectively. Creatine kinase levels greater than 2000 units/L could be a warning sign of compartment syndrome in an anesthetized patient [33]. We did not find a significant difference between the groups’ CK levels. However, it was greater than 2000 units/L in rats exposed to high pressure that would cause ACS for 90 min. This indicated that CK started to increase not in the first 45 min of ACS but in 90 min, which was consistent with other findings of our study.

For instance, IMA increased with reasonable sensitivity and specificity in critical limb ischemia although its role in assisting the diagnosis of ACS has not been fully established [34]. Similar to the CK values, the IMA values measured at the 90th minute of the high ICP were high in our study and no significant change was found on the 45 min of increased ICP. However, it should be noted here that low albumin values affect the IMA values [35].

Methods such as near-infrared spectroscopy (NIRS), pH and glucose values of the compartment, and USG and Doppler flowmetry are promising for the future for the diagnosis of ACS in the early period.

Studies have shown that low tissue oxygenation levels are associated with increased IMP [36]. A sudden decrease in tissue oxygenation in patients with ACS has been demonstrated by the NIRS method, which measures tissue oxygenation using hemoglobin saturation and provides an indirect measurement of the perfusion status of the affected compartment [37], but the reliability of NIRS in the traumatic limb remains unclear and clinical studies are needed to fully define its role in the diagnosis of ACS [38]. However, the possibility of measurement error in trauma patients when applied above the bruised skin color and on subcutaneous hematoma areas, and the limited ability to reach deep compartments that are risky for the development of ACS, are sources of concerns about the consistency of NIRS [39].

Ultrasound has been used to monitor the status of localized perfusion [2]. However, the evaluation of the variations in compartment geometry and echogenicity increased the echogenicity in the images due to the high pressure in the ACS and the relationship and repeatability were poor [40].

Acute electrodiagnostic methods are rarely used in the diagnosis of ACS. However, the presence of a nerve injury in the part where the ACS develops and the inability of the muscles in this part to be activated may cause false positives. Moreover, if electrodiagnostic methods are used on an affected muscle and stimulate the proximal nerve damage, it cannot distinguish between muscle necrosis and primary nerve damage, which is a complete conduction block or axonal loss in the muscle [39].

It has been shown that laser Doppler flowmetry and scintigraphy are effective methods in the evaluating of local perfusion and both techniques are applicable in the diagnosing of ACS [41,42]. However, time-consuming scans, lack of specificity of the traumatized limb, and the inability of repeated examinations make scintigraphy inappropriate in the diagnosing of ACS. The use of laser Doppler flowmeters in the diagnosing of ACS has not yet been evaluated.

In an experimental study, it was showed that the glucose concentration and oxygen level in the muscle were significantly lower in the 15th minute after the ACS occurred [22]. Although this method, which can detect changes in ACS, has great potential, it is stated that the technology of glucose sensors should be developed more in order to be used on humans. Because glucose sensors cannot provide readings at values less than 40 mg/dL, several hours are required before the calibration process can be performed and accurate glucose data can be measured. In addition, glucose threshold values determined to detect compartment syndrome may not be exactly compatible for humans, and clinical studies are required to determine the threshold values [22].

It has been suggested that the surface pH of skeletal muscle is a sensitive indicator of peripheral muscle blood flow and gives better results than continuous measurement of IMP in the diagnosis of ACS [2,4,43]. However, it is necessary to develop probes to measure the muscle’s pH and conduct clinical studies on humans because, with today’s methods, pH analysis is both time-consuming and repetitive [2].

## 5. Limitations

In terms of the animal model used, our sample size was small, the saline infusion model used has differences from actual trauma, and the generalizability of the results was limited. In addition, the level of biochemical markers and morphological changes at the reversible and irreversible stages of ACS-related cell damage should be accurately determined and correlated for cellular metabolic markers to be directly associated with ACS.

## 6. Conclusions

If ACS is not diagnosed early, it is a condition that threatens the patient’s life and limb. Despite the need for early diagnosis and extensive research, a reliable and objective method to diagnose it at an early stage has not been developed yet. Although some promising methods have been developed with experimental studies, randomized controlled studies are needed to evaluate the potential and effectiveness of these methods. According to our study, TOS seems to be able to detect ACS at the earliest stage. However, it should be emphasized that in-compartment pressure measurement is still the gold standard method used for ACS diagnosis. However, it should be kept in mind that in-compartment pressure measurement may cause unnecessary fasciotomy in cases where it has no specificity for diagnosis and is the only criterion considered in the diagnosis of ACS.

## Figures and Tables

**Table:** The results of biochemical markers.

Serum parameters	Groups	Median ± SD (min-max)	*x* *^2^*	*p*
**IMA (ng/mL)**	**SG1**	212.24 **±** 155.42 (124.04–554.60)^a^	7.000	0.095^ac^
	**SG2**	406.33 ± 248.53 (212.70–895.25)^b^		**0.030**
	**CG**	144.25 ± 31.72 (106.26–169.26)^c^		**0.020** bc
**TAS (mmol/L)**	**SG1**	1.43 ± 0.28 (1.35–2.10)	3.820	0.167^ac^
	**SG2**	1.60 ± 0.10 (1.47–1.77)		0.148
	**CG**	1.63 ± 0.02 (1.61–1.66)		0.548^bc^
**TOS (μmol/L)**	**SG1**	52.80 ± 15.45 (44.40–88.00)^a^	11.125	**0.048** ac
	**SG2**	127.30 ± 30.07 (83.58–171.70)^b^		**0.004**
	**CG**	41.80 ± 4.06 (37.40–45.40)^c^		**0.024** bc
**ALT (U/L)**	**SG1**	51 ± 7.47 (43.00–62.00)^a^	7.945	0.095^ac^
	**SG2**	107.50 ± 154.03 (54.00–464.00)^b^		0.091
	**CG**	70.00 ± 10.44 (53.00–72.00)^c^		0.262^bc^
**AST (U/L)**	**SG1**	119 ± 20.07 (96.00–146.00)^a^	11.370	0.095^ac^
	**SG2**	302.0 ± 334.77 (202.00–944.00)^b^		**0.003**
	**CG**	190.00 ± 40.85 (123.00–197.00)^c^		**0.021** bc
**CK (U/L)**	**SG1**	804 ± 1310.49 (470.00–3910.00)^a^	5.058	0.714^ac^
	**SG2**	2745.0 ± 1962.05 (842.00–6222.00)^b^		0.08
	**CG**	873.00 ± 230.47 (772.00–1212.00)^c^		0.095^bc^
**Neopterin (ng/mL)**	**SG1**	29.03 ± 4.96 (18.03–32.06)^a^	8.900	0.262^ac^
	**SG2**	36.30 ± 3.83 (28.33–38.68)^b^		**0.012**
	**CG**	24.13 ± 3.00 (21.96–27.89)^c^		**0.016** bc

SG1: Study group 1.

SG2: Study group 2.

CG: Control group.

ac When comparing SG1with the CG.

bc When comparing SG2 with the CG.

Median ± SD: Median ± standard error; IMA: ischemia-modified albumin; TAS: total antioxidant status; TOS: total oxidant status; AST: aspartate transferase; ALT: alanine aminotransferase; CK: creatine kinase
